# Mechanisms and Management Strategies of Hepatocarcinogenesis Driven by Chronic Hepatitis B Comorbid with Type 2 Diabetes

**DOI:** 10.3390/microorganisms14040853

**Published:** 2026-04-10

**Authors:** Qin Lou, Jiarong Cai, Jianhua Yin

**Affiliations:** 1 Department of Epidemiology, Naval Medical University, Shanghai 200433, China; 18769397166@163.com (Q.L.); jiarongcai6@163.com (J.C.); 2 Key Laboratory of Biological Defense, Ministry of Education, Naval Medical University, Shanghai 200433, China; 3 Shanghai Key Laboratory of Medical Bioprotection, Naval Medical University, Shanghai 200433, China

**Keywords:** chronic hepatitis B, type 2 diabetes, comorbidity, hepatocellular carcinoma, mechanisms, management strategies

## Abstract

Chronic hepatitis B (CHB) and Type 2 diabetes (T2DM) are major independent risk factors for Hepatocellular carcinoma (HCC). The bidirectional promotion between T2DM and CHB forms the biological basis for their synergistic carcinogenic effect. T2DM mainly accelerates the progression of CHB through mechanisms such as metabolic disorders, oxidative stress, chronic inflammation, and immunosuppression; CHB promotes the development of T2DM mainly through liver damage leading to dysfunction of the central glucose metabolism, HBx-driven gluconeogenesis, inhibition of the insulin signaling pathway, and potential β-cell damage. In comorbid conditions, these mechanisms intertwine to form a vicious cycle across four key aspects: metabolic and lipid disorders, activation of carcinogenic pathways, oxidative stress, and amplification of chronic inflammation, significantly accelerating the hepatocarcinogenesis. Regarding management strategies, we adopt the concept of three-level prevention, integrate various management plans and combine emerging drug therapies. We thus propose the establishment of a management strategy centered on “liver and glucose co-management” with multi-faceted joint control. This review aims to summarize the latest evidence on the mechanisms and management strategies by which the comorbidity of T2DM and CHB promotes the development of HCC, providing a theoretical basis for research on the mechanisms of this comorbidity and population-level HCC prevention strategies.

## 1. Introduction

### 1.1. Disease Burden

Liver cancer is the sixth most common cancer worldwide and the third leading cause of cancer-related mortality. It is projected that the global number of newly diagnosed liver cancer cases will rise from 870,000 in 2022 to 1.52 million by 2050. Among these cases, hepatocellular carcinoma (HCC) accounts for approximately 80% of all liver cancer diagnoses [1]. Over the past two decades, the global incidence and mortality rates of liver cancer have increased by 53.7% and 48.0%, respectively. This upward trend is particularly pronounced in high-incidence regions such as Asia and Africa [2]. According to assessments of the global burden of HCC, cases of hepatitis B virus-associated HCC are predominantly distributed in China, South Korea, India, Japan, and Thailand [3]. The etiology of HCC is complex, involving both traditional risk factors such as viral hepatitis infections, aflatoxin exposure, and alcohol consumption, as well as metabolic risk factors like obesity and diabetes, which have garnered increasing attention in recent years [4]. With the rising prevalence of metabolic disorders such as metabolic syndrome, obesity, type 2 diabetes mellitus (T2DM), and metabolic dysfunction-associated steatotic liver disease (MASLD), their synergistic effects may become a primary etiological factor for HCC [5]. In China, liver cancer ranks fourth in incidence and second in mortality among cancers. In 2020, there were approximately 410,000 new cases of liver cancer domestically, accounting for 45.3% of the global total. Among these, HBV infection is the predominant risk factor, with approximately 69.9% of HCC patients in China having a background of HBV infection [6].

Diabetes mellitus (DM) is a chronic metabolic disorder characterized by hyperglycemia. Based on its pathogenesis, which stems from either absolute insulin deficiency or insulin resistance (IR), the disease is primarily classified into type 1 and type 2. T2DM is accompanied by IR and hyperinsulinemia, which are insufficient to maintain normal glucose metabolism [7,8]. In 2022, the number of diabetes patients worldwide was 828 million, of which over 95% had T2DM [9]. In 2021, there were approximately 145 million diabetes patients in China, with an age-standardized prevalence of about 10.6% [10].

Chronic hepatitis B (CHB) is one of the leading causes of cirrhosis, HCC, and liver-related deaths worldwide [11]. By 2022, the global HBV infection rate was about 3.2%, with approximately 258 million people testing positive for HBsAg [12]. Among them, China bears the heaviest burden of hepatitis B in the world, with approximately 75 million people infected with HBV, accounting for roughly one-third of the global total. Nevertheless, over the past three decades, China has made significant progress in controlling HBV infections, with the hepatitis B vaccination rate reaching 99.6% [13].

### 1.2. Interactive Correlation

Both CHB and T2DM are independent risk factors for HCC. Based on the results of multiple meta-analyses and large cohort studies, the risk ratio (RR/HR) for diabetic patients developing hepatocellular carcinoma (HCC) ranges roughly from 1.86 to 7.52 [14]. T2DM is associated with an increased risk of liver cancer, and there is a correlation between HbA1c and the risk of HCC, with an aHR of 1.37 (1.08–1.74) [15]. Compared with T2DM, CHB leads to a significantly higher risk of developing HCC, approximately 10 to 30 times that of the non-CHB population [16].

Increasing evidence suggests that CHB and T2DM have a significant mutually promoting effect, which constitutes the biological basis for the heightened risk in comorbidity. Several population-based studies have shown that the incidence and prevalence of T2DM are significantly higher in individuals with chronic HBV infection compared to the general population [17]. A prospective cohort study of 55,520 Chinese adults found that different HBV infection statuses were associated with a gradient risk of developing T2DM: compared with uninfected individuals, those who had recovered from HBV infection had the highest risk of new-onset T2DM (HR = 1.22), while current CHB patients also had a significant risk (HR = 1.18) [18]. Research has also shown that among HBsAg-positive individuals, high levels of serum HBV DNA are independently associated with an increased risk of T2DM [19].

In terms of comorbidity risk, two meta-analyses have demonstrated that T2DM significantly increases the risk of HCC in CHB patients, with risk ratios ranging from 1.26 to 1.37 [20,21]. A study based on the GBD 2019 database evaluated the burden of liver cancer caused by the HBV-T2DM comorbidity. The results indicated that from 1990 to 2019, both the age-standardized prevalence and years of life lost due to disability-adjusted life years of liver cancer caused by HBV-T2DM comorbidity showed an upward trend in 16 regions worldwide [22]. Research indicates that T2DM is an independent risk factor for liver fibrosis in CHB patients [23], and is also associated with an increased risk of CHB-related HCC [24]. The long-term impact of comorbidities cannot be ignored. In patients with CHB combined with T2DM, poor glycemic control is significantly associated with the severity of liver fibrosis. Optimizing blood glucose management and the use of hypoglycemic drugs may slow the progression of liver fibrosis [25]. Evidence from cohort studies is summarized (Table 1).

## 2. The Mechanism of Carcinogenic Interaction Between T2DM and CHB Comorbidity

### 2.1. T2DM Aggravates CHB Progression

The presence of T2DM exacerbates liver injury in CHB and promotes the progression of liver fibrosis and HCC. This process is likely mediated by complex mechanisms involving lipotoxicity, oxidative stress, and inflammatory pathways induced by hyperglycemia and hyperinsulinemia [14,26].

Metabolic disorders and lipid accumulation: T2DM drives elevated serum free fatty acid levels through hyperinsulinemia, thereby promoting HBV replication. Under normal physiological conditions, insulin can inhibit lipolysis in adipose tissue. However, in the context of T2DM, the liver and peripheral tissues develop IR, leading to the failure of insulin’s inhibitory effect on lipolysis. Meanwhile, hepatic de novo lipogenesis in the liver is abnormally elevated, causing a large influx of free fatty acids into the liver. This process exacerbates hepatic lipid accumulation and steatosis, creating a favorable microenvironment for HBV replication [27,28]. At the same time, IR can lead to dysfunction of visceral adipose tissue, characterized by increased fatty acid oxidation and release, and altered secretion profiles of inflammatory factors (such as elevated levels of TNF-α, IL-6, and leptin), thereby exacerbating liver inflammation and fibrosis, and promoting the malignant progression of CHB [29].

Metabolic dysfunction-associated steatotic liver disease (MASLD)/metabolic dysfunction-associated steatohepatitis (MASH): In the context of MASLD/MASH, the pro-tumorigenic mechanisms of T2DM in HCC have become a major focus in hepatology and metabolism. IR acts as the central mediator connecting diabetes to the progression of MASLD-associated HCC. Specifically, chronic hyperinsulinemia directly promotes abnormal proliferation of hepatocytes via activating pro-proliferative signaling pathways including PI3K/Akt/mTOR [30,31]. Meanwhile, IR induces profound disturbances in hepatic lipid metabolism. Compromised hepatic gluconeogenesis coincides with excessive activation of de novo lipogenesis, generating lipotoxic intermediates (e.g., ceramides, diacylglycerols) that elicit sustained oxidative stress, endoplasmic reticulum stress, and DNA damage. These events collectively drive the transition from hepatic steatosis to MASH and eventually to HCC [32,33]. Notably, a substantial proportion of MASLD-related HCC develops in the absence of cirrhosis, differing from the classic viral hepatitis–cirrhosis–HCC sequence. This indicates diabetes-associated metabolic reprogramming may directly promote hepatocarcinogenesis via fibrosis-independent pathways [34]. This provides a novel perspective for the therapeutic strategy of liver and glucose co-management.

Oxidative stress-mediated liver injury: Under T2DM conditions, persistent hyperglycemia and dietary intake lead to a large accumulation of advanced glycation end products (AGEs), causing oxidative stress and mitochondrial dysfunction. In this process, impaired glucose metabolism and dysfunction in the mitochondrial respiratory chain lead to increased electron leakage, thereby generating a large amount of reactive oxygen species (ROS), which directly attack hepatocytes, induce DNA damage, further exacerbate oxidative stress, and directly result in hepatocyte injury and liver fibrosis. At the same time, obesity or dyslipidemia associated with T2DM can exacerbate fatty acid oxidation, increasing the levels of free fatty acids, further worsening mitochondrial dysfunction and increasing ROS production, thereby creating a vicious cycle [14,35,36]. In addition, oxidative stress not only causes metabolic liver damage, but the gene mutations it induces, protein dysfunction, and persistent liver cell injury with impaired repair functions all create conditions for the malignant transformation of hepatocytes in the context of HBV infection, directly driving the progression of liver fibrosis and HCC in CHB [37].

Chronic inflammation and signaling pathway activation: Oxidative stress can lead to chronic inflammation and immunosuppression, and by activating pathways such as NF-κB, it causes a massive release of inflammatory factors like TNF-α and IL-6, creating a persistent pro-inflammatory liver microenvironment. This inflammatory environment not only directly exacerbates liver damage and fibrosis, but also creates conditions for the persistent replication and latency of HBV by inhibiting effective antiviral immune responses. Of particular importance, inflammatory factors represented by TNF-α can directly alter the transcriptional activity of pro-inflammatory genes and others through epigenetic mechanisms such as regulating histone modifications and DNA methylation. This inflammation-triggered epigenetic reprogramming can stabilize and amplify the expression programs of pro-inflammatory genes, thereby maintaining and exacerbating chronic inflammation at the molecular level [37].

Immune dysfunction: Chronic hyperglycemia, inflammation, and oxidative stress associated with T2DM can lead to widespread immune dysfunction, including weakened innate immune responses, impaired T cell function, and the promotion of immune exhaustion. This systemic immunosuppressive state directly weakens the body’s ability to specifically clear HBV, leading to ineffective control of the virus and continuous replication. At the same time, imbalances in immunometabolism (such as dysfunction of immune cells in insulin-sensitive tissues) can further exacerbate local liver inflammation and oxidative stress [27,28]. Studies have shown that the immune system function of patients with T2DM may be affected, making the body more susceptible to infections. Immune cell infiltration, inflammation, and oxidative stress can promote metabolic disorders in insulin-sensitive tissues, and the resulting immune system impairment and metabolic imbalance also increase patients’ susceptibility to various pathogens, including HBV [38].

### 2.2. CHB Disrupts Glucose Homeostasis and Promotes T2DM

Liver damage and dysfunction of metabolic centers represent one of the core mechanisms by which chronic HBV carriers exhibit a higher risk of developing T2DM. Essentially, HBV infection causes liver damage that disrupts the liver’s regulation of glucose metabolic homeostasis, leading to glucose metabolism disorders [35]. Specifically, hepatocellular damage, necrosis, and subsequent inflammation reduce the activity of enzymes involved in glycogen synthesis (e.g., hexokinase, glycogen synthase), directly impairing glucose uptake and utilization, resulting in elevated blood sugar levels [19]. On the other hand, HBV infection can lead to a downregulation of insulin receptor levels and impaired activity in liver cells, while the liver’s ability to clear insulin antagonists (e.g., inflammatory factors, free fatty acids) decreases, leading to their elevated circulating levels and further antagonism of insulin action. Collectively, these alterations establish a state of IR, prompting pancreatic β cells to compensatorily secrete more insulin, ultimately leading to hyperinsulinemia and an increased risk of T2DM [39].

Molecular Mechanism by which HBx Promotes Hepatic Glucose Metabolism Disorders: The HBx protein encoded by HBV can abnormally enhance hepatic gluconeogenesis by interfering with the host’s gluconeogenesis regulatory network. Studies have shown that HBx can activate the NO/JNK signaling pathway, thereby promoting the nuclear translocation and transcriptional activity of the transcription factor FOXO1, significantly upregulating the gene expression of key gluconeogenic enzymes—phosphoenolpyruvate carboxykinase (PEPCK) and glucose-6-phosphatase (G6Pase). Even in a non-fasting state, this mechanism continues to drive excessive hepatic glucose production, leading to hepatic hyperglycemia, which constitutes one of the important pathological bases for the development of T2DM. This molecular pathway has been validated in HBx transgenic animal models, showing increased expression of hepatic gluconeogenic enzymes and elevated fasting blood glucose levels [36].

Inhibition of the insulin signaling pathway: Suppressor of Cytokine Signaling (SOCS) is a class of proteins that negatively regulate cytokine signaling and insulin signaling pathways. They can use feedback to regulate through the JAK/STAT pathway or directly interfere with the phosphorylation of insulin receptor substrates (IRS), thereby inducing IR. Hepatitis viruses can induce the expression of SOCS, thereby interfering with insulin signal transduction and promoting IR. STAT3 is an important member of the signal transducer and activator of transcription family, mediating various inflammation-related signaling pathways. Hepatitis viruses can drive interferon-γ (IFN-γ)-mediated β-cell apoptosis through the STAT3/IL-6/S100A8/A9 cascade: Persistent expression of HBx activates the JAK/STAT3 signaling pathway in the liver, inducing the expression of inflammatory factors S100A8/A9; S100A8/A9, acting as ligands, circulate through the blood to the pancreas, bind to RAGE receptors on islet β-cells, and activate the p38 MAPK pathway, thereby stimulating natural killer (NK) cells to produce IFN-γ; IFN-γ directly induces islet β-cell apoptosis through synergistic pro-inflammatory mechanisms (such as activating the NF-κB/Bcl2 pathway), leading to reduced insulin secretion. At the same time, STAT3 pathway-driven systemic inflammation further exacerbates IR, together increasing the risk of developing T2DM [29,40].

Pancreatic β-cell damage: Studies have shown that elevated serum HBV-DNA levels are associated with an increased risk of T2DM, along with higher fasting blood glucose and glycated hemoglobin levels. This association may involve multiple potential mechanisms. Hepatocellular inflammatory necrosis can impair the liver’s ability to clear insulin and glucagon, leading to elevated levels of both in the circulation; in the context of IR, a relatively high glucagon state may further promote hepatic glucose output, worsening glucose metabolism disorders, which in turn decreases glucose tolerance and exacerbates IR. HBV DNA or HBsAg is not only present in hepatocytes, but also in pancreatic tissue and pancreatic juice. HBV replication in the pancreas can trigger a local inflammatory and immune response, leading to immune-mediated β-cell damage, suggesting that HBV may directly, or through immune mediation, damage pancreatic β-cells. This may serve as one of the important mechanisms underlying the development of T2DM in HBV-infected individuals, particularly those with high serum HBV DNA levels [19,41,42,43,44].

### 2.3. Synergistic Mechanisms of T2DM and CHB in HCC Development

#### 2.3.1. Interactive Carcinogenesis Network

**Figure 1:**
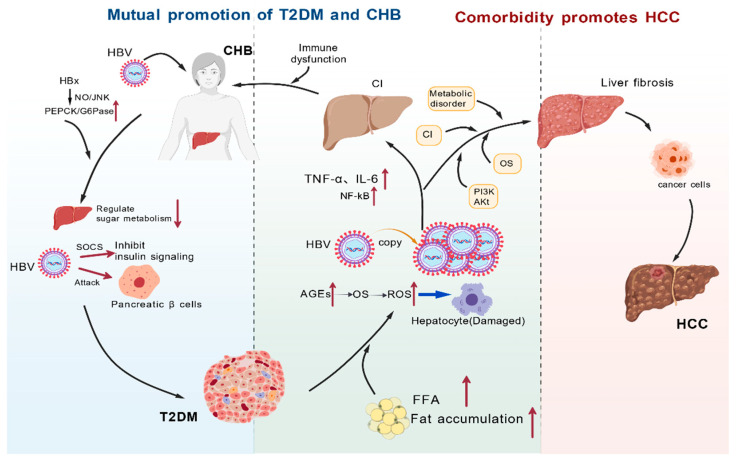
The interaction between CHB and T2DM in comorbidity and their synergistic promotion of HCC. Arrows indicate activation/upregulation (↑) or inhibition/downregulation (↓). HBV: hepatitis B virus; CHB: chronic hepatitis B; HBx: hepatitis B protein X; T2DM: Type 2 diabetes; HCC: hepatocellular carcinoma; PEPCK/G6Pase: Phosphoenolpyruvate carboxykinase/Glucose-6-phosphatase; NO/JNK: Nitric Oxide/c-Jun N-terminal Kinase pathway; SOCS: Suppressor of Cytokine Signaling; FFA: fatty acids; AGEs: Advanced Glycation End Products; OS: Oxidative Stress; ROS: reactive oxygen species; TNF-α: Tumor Necrosis Factor-alpha; IL-6: interleukin-6; NF-κB: Nuclear Factor Kappa-B pathway; PI3K/Akt: Phosphoinositide 3-Kinase/Protein Kinase B pathway; CI: Chronic Inflammation.

Currently, there is little research exploring the synergistic carcinogenic mechanisms of T2DM and CHB, but their synergistic effect may stem from the interaction and amplification of several key pathways: first, metabolic and lipid disorders; second, activation of oncogenic signaling pathways; third, exacerbation of oxidative stress; and fourth, amplification of chronic inflammatory cascades. In the context of comorbidities, the aforementioned pathways intertwine and synergistically enhance each other, collectively forming a dynamic cooperative pro-cancer network, ultimately leading to uncontrolled unstable proliferation of hepatocytes, evasion of apoptosis, formation of a tumor immune microenvironment, and significantly increasing the risk of HCC. A summary of the core molecular mechanisms of the above interactive carcinogenic network is shown in Figure 1.

Metabolic and lipid disorders: T2DM-related hyperinsulinemia and IR promote peripheral fat lipolysis and hepatic de novo lipogenesis, leading to abnormal accumulation of free fatty acids in the liver, causing significant lipid metabolism disorders, which in turn promote HBV replication [27,28]; IR mediates the increase in TNF-α, IL-6, and leptin levels [29], promoting the progression of CHB to fibrosis and HCC. At the same time, liver damage caused by CHB can impair the liver’s ability to regulate glucose metabolism, leading to systemic glucose metabolism disorders and exacerbated IR [35], promoting the progression of T2DM. The interaction between T2DM and CHB forms a vicious cycle, increasing the risk of CHB progressing to HCC.

Activation of carcinogenic signaling pathways: Hyperinsulinemia associated with T2DM can increase the bioavailability of insulin-like growth factor-1 (IGF-1), thereby activating the PI3K/AKT/mTOR signaling pathway. This pathway plays a role in promoting hepatocyte proliferation and inhibiting apoptosis, and it provides key growth signals for abnormal hepatocyte proliferation and malignant transformation in the context of HBV infection. It is considered a crucial pathway for liver cancer associated with fatty liver caused by metabolic disorders [14]. The signal transduction mediated by IGF-1 binding to its receptor (IGF-1R) not only participates in regulating the pluripotency and differentiation of stem cells during embryonic development, promoting normal cell proliferation and tissue regeneration, but once this signaling system becomes imbalanced, it can promote cancer cell proliferation and trigger tumor-related reprogramming processes in tissues such as the liver [29]. HBx can also activate the PI3K/Akt pathway, further enhancing protein synthesis and cell metabolism, and inhibiting apoptosis [45,46]. In the comorbid state of T2DM and CHB, the two may synergistically activate the aforementioned carcinogenic signaling pathways, significantly accelerating the development of HCC.

Exacerbation of oxidative stress: Oxidative stress is a known cause of vascular damage caused by diabetes and can contribute to cancer by inducing DNA oxidative damage and gene mutations. The hyperglycemic state in T2DM can trigger mitochondrial dysfunction through AGEs, produce a large amount of ROS, and exacerbate oxidative stress levels [37]. HBV proteins can also mediate intracellular oxidative stress by acting through multiple organelles, increasing ROS production, inhibiting antioxidant defenses, and amplifying a vicious cycle, ultimately leading to hepatocellular carcinogenesis. The core mechanisms mainly include the following points: 1. Endoplasmic reticulum (ER) stress induction: HBsAg and HBcAg abnormally accumulate in the ER, activating the unfolded protein response (UPR). The UPR leads to the release of hydrogen peroxide, significantly increasing reactive oxygen species (ROS) levels, and triggering DNA damage and genomic instability. 2. Mitochondrial dysfunction: HBx protein targets mitochondria and inhibits electron transport chain activity, resulting in impaired respiratory function, excessive production of superoxide anions, and DNA oxidative damage such as 8-oxoguanine. 3. Inhibition of the antioxidant system: Suppression of antioxidant signaling pathways such as Nrf2/ARE reduce the activity of antioxidant enzymes like catalase. 4. Formation of a pro-carcinogenic environment: Activation of transcription factors such as NF-κB and STAT3 promote the release of inflammatory factors and stellate cell activation, collaboratively driving the malignant transformation of cells [47]. Comorbid conditions may exacerbate oxidative stress levels, synergistically enhancing carcinogenic effects.

Amplification of the chronic inflammation cascade: Oxidative stress caused by T2DM deeply triggers chronic inflammation and immunosuppression, activates pro-inflammatory signaling pathways, and leads to a massive release of inflammatory factors (e.g., NF-κB), creating a persistent pro-inflammatory microenvironment [37]. Oxidative stress caused by CHB can also activate the NF-κB pathway. In addition, CHB can trigger immune dysregulation, leading to dysfunction of various immune cells (such as CD4^+^ T cells, NK cells, macrophages, etc.) and driving persistent liver inflammation [48]; there are also bystander CD8^+^ cells in the liver that belong to the CD8^+^ cell subset, which can also cause inflammation and liver toxicity [49]. When T2DM and CHB coexist, a vicious cycle of viral inflammation–metabolic deterioration–inflammation amplification is formed. Their interaction leads to a continuous escalation of the inflammatory cascade, exacerbating organ damage and promoting the progression to HCC in patients with comorbidities.

The high invasiveness and therapeutic resistance of HCC are closely related to its immunosuppressive tumor microenvironment (TME). Neutrophil extracellular traps (NETs), which are web-like structures composed of chromatin and granular proteins released during NETosis, have become a major driver of inflammation in the HCC TME. NETs actively promote tumor progression by physically capturing circulating tumor cells, remodeling the extracellular matrix, stimulating angiogenesis, and facilitating immune evasion. As an emerging inflammatory bridge between innate and adaptive immunity, NETs promote the differentiation of initial CD4^+^ T cells into regulatory T cells (Tregs) by inducing metabolic reprogramming, thereby forming an immunosuppressive microenvironment. Meanwhile, NETs can induce Th17 cell differentiation; IL-17A secreted by these Th17 cells further activates neutrophils and promotes NETosis, creating a positive feedback amplification loop of “NETs → Th17 differentiation→IL-17A→enhanced NETs,” continuously amplifying liver inflammation. IL-1β, as a key inducer of NETosis, further amplifies inflammatory signals through activation of the inflammasome pathway. In the context of metabolic dysfunction-associated steatohepatitis (MASH)/non-alcoholic steatohepatitis (NASH), hyperglycemia, free fatty acids, and lipotoxicity can all induce NETosis. NETs promote liver fibrosis by activating hepatic stellate cells, exacerbate inflammation through NLRP3 inflammasome-mediated monocyte recruitment, and support tumor growth by promoting tumor angiogenesis. Recent multi-omics studies have found that SPP1^+^ M2 macrophages and NETs^+^ neutrophils preferentially colocalize at the tumor-stroma interface. Through interactions via OPN(SPP1)-CD44/integrin and ICAM1-β2-integrin axes, they activate downstream FAK-PI3K-Akt and NF-κB/MAPK pathways, jointly constructing a high-risk NETosis microenvironment. These mechanisms suggest that NETs are a key inflammatory bridge that synergistically promotes liver cancer development in patients with T2DM combined with CHB/MASH [50].

#### 2.3.2. Epigenetic Mechanism

Epigenetic regulation of the virus: HBV cccDNA binds to histones in the host cell nucleus to form minichromosomes, and its transcriptional activity is strictly regulated by epigenetic modifications. This is the molecular basis for persistent HBV infection and carcinogenesis. Histone acetylation is key to maintaining efficient cccDNA transcription. HBx facilitates histone acetylation by recruiting histone acetyltransferases (such as CBP/p300) to the cccDNA minichromosome, thereby maintaining an active transcriptional state and driving the continuous expression of viral proteins (including HBx itself), which promotes malignant transformation of hepatocytes. Conversely, in the absence or low expression of HBx, cccDNA exhibits a repressive chromatin state characterized by histone hypoacetylation and H3K9 methylation enrichment, along with the recruitment of HDAC1, the methyltransferase SETDB1, and the heterochromatin protein HP1, leading to markedly reduced viral transcription and weakening the virus’s oncogenic activity. In addition, HBx can interact with DNA methyltransferases (DNMTs) to regulate DNA methylation patterns of both cccDNA and host chromatin, thereby affecting viral transcription and host gene expression and further contributing to liver cancer development [51,52].

Epigenetic Remodeling Induced by T2DM: Hyperglycemia, as an important factor affecting liver metabolism, can trigger persistent epigenetic changes. High blood glucose can affect the expression and activity of DNA methyltransferases (DNMTs) and TET demethylases, causing disruption of the methylation patterns in the promoter regions of liver metabolic genes, forming stable epigenetic phenotypes. Hepatic IR is not merely a defect in signaling pathways but a result of overall epigenomic reprogramming. Meanwhile, IR can in turn reinforce abnormal epigenetic modifications, forming a positive feedback loop. Under the state of IR, the expression and nuclear localization of insulin signaling downstream transcription factors (such as FoxO1) are abnormal, which recruits epigenetic modifying enzymes (such as HDAC, EZH2), leading to an imbalance in histone methylation/acetylation. IR directly results in the epigenetic stabilization of the liver metabolic gene expression profile: genes involved in gluconeogenesis and lipid synthesis remain persistently activated, while genes related to insulin sensitivity are silenced, making it difficult to reverse the state of IR. The accumulation of lipid intermediates such as free fatty acids (FFAs), diacylglycerol (DAG), and ceramides triggers hepatocellular lipotoxicity, which is an important metabolic stress source inducing epigenetic remodeling. Lipotoxicity can upregulate HDACs and the H3K27me3 modifying enzyme EZH2, inhibit key genes in the insulin signaling pathway, and simultaneously activate chromatin accessibility of pro-inflammatory and lipogenic genes. Oxidative stress inhibits TET enzyme activity, leading to abnormal increases in genomic DNA methylation; it also affects histone demethylases (such as the JMJD family), causing disordered histone methylation modifications. Chronic metabolic inflammation activates NF-κB, AP-1, and other transcription pathways through pro-inflammatory factors such as TNF-α and IL-6, recruiting epigenetic modification complexes to remodel hepatocyte chromatin. Pro-inflammatory signaling factors can induce abnormal histone modifications and DNA methylation changes, maintaining continuous activation of inflammation-related genes and mutually promoting IR [53,54].

Non-coding RNA/Imprinting Site Regulation: The non-coding RNA regulatory networks and abnormal imprinting mechanisms linking metabolic disorders to tumorigenesis are numerous, involving multiple aspects such as glycolysis reprogramming, mitochondrial metabolism inhibition, lipid and glutamine metabolism dysregulation, core transcription factor mediation, epigenetic positive feedback loops, and imprinting loss. miRNAs (such as miR-143) can directly inhibit glycolytic enzymes HK2, PKM2, and GLUT1, or lncRNAs/circRNAs can act as ceRNA sponges to absorb these miRNAs, leading to increased glucose uptake and lactate production (Warburg effect), providing tumors with energy and synthetic precursors, and directly driving proliferation and invasion [55]. NCRNAs (such as NRF1) downregulate genes related to oxidative phosphorylation (OXPHOS), weakening mitochondrial function, increasing ROS, and shifting metabolism from respiration to glycolysis [56]; ROS causes DNA damage and genomic instability, inducing malignant transformation. ncRNAs upregulate key enzymes of fatty acid synthesis, FASN and ACC, promoting excessive lipid synthesis and membrane remodeling; abnormal lipid metabolism activates oncogenic signals such as PI3K/Akt, accelerating tumor initiation and metastasis. ncRNAs regulate HIF-1α and c-Myc, comprehensively activating glycolysis, glutamine metabolism, and biosynthesis, or inhibit p53 (tumor-suppressive metabolic regulation), shifting metabolism entirely toward a pro-cancer state, initiating and sustaining metabolic reprogramming. lncRNAs recruit epigenetic complexes such as PRC2 to silence metabolism-related tumor suppressor genes; metabolites (SAM/α-KG) in turn modify ncRNA expression, forming a metabolism–epigenetics–ncRNA positive feedback loop that stabilizes a pro-cancer metabolic state. Loss of imprinting (LOI) of IGF2/H19 and others leads to dysregulated lncRNA/miRNA expression, disrupting insulin/growth factor metabolic homeostasis; imprinting imbalance becomes a bridge for the transformation from metabolic disease to tumorigenesis, driving carcinogenesis [57].

Summary of the above-mentioned mechanisms (Table 2).

## 3. Management Strategy

Currently, there are no systematic clinical practice guidelines for the comorbidity of CHB and metabolism-related diseases [58]. Given that comorbidities substantially contribute to the global disease burden and elevate the risk of HCC, it is critical to establish a management strategy centered on “liver–glucose co-therapy” featuring comprehensive and integrated control. This section adopts the concept of tertiary prevention, integrates various management programs, and combines emerging drug therapies to summarize the following comprehensive management strategies.

### 3.1. Primary Prevention

Primary prevention aims to reduce the risk of co-occurrence of CHB and T2DM. Patients with T2DM should be prioritized for receiving the hepatitis B vaccine. The Hepatitis B vaccine prevents HCC by inducing anti-HBs-mediated active immunity to block chronic HBV infection. It abrogates viral DNA integration, HBx oncoprotein expression, and the chronic inflammation–necrosis–regeneration cycle [59]. Conversely, chronic HBV infection disrupts glucose metabolism by enhancing hepatic gluconeogenic signaling (glucagon/cAMP/PKA/CREB pathway) via the small hepatitis B surface antigen (SHBs) [60]. Vaccination eliminates this metabolic disturbance and reduces diabetes risk by preventing chronic HBV infection [61].

According to CDC recommendations, vaccination should include infants, children, adolescents, all adults under 59, and adults over 60 with risk factors [62,63]. In addition, patients with diabetes mellitus (DM) may have an increased risk of HBV infection due to the need for frequent blood glucose monitoring and insulin injections [64]; therefore, strictly adhering to proper blood glucose monitoring and syringe sterilization is also an important aspect of hepatitis B prevention strategies. At the same time, it is important to promote a healthy lifestyle among the HBV population to prevent T2DM, such as regular exercise, a healthy diet, and smoking cessation. It is recommended that adults aged 18 and over should complete at least 150 min of moderate-intensity activity each week [65]. Multiple randomized trials have confirmed that the risk of T2DM can be reduced by 30–60% through exercise, diet, or a combination of both; for every kilogram of weight lost, the risk of developing T2DM can decrease by 16% accordingly [66].

### 3.2. Secondary Prevention

The core of secondary prevention is to carry out systematic screening and early diagnosis for high-risk populations, shifting the focus of liver cancer prevention and control to an earlier stage. The hidden prevalence of HBV infection is quite common, with many infected individuals showing no symptoms for a long time. This latent period can last for decades, or even a lifetime. A study by Cui et al. showed that [67], in 2019, only 10.3% of people with chronic HBV infection worldwide were diagnosed, and only 21.8% of those diagnosed received treatment, resulting in an overall treatment coverage rate of just 2.2%. This situation is common and may delay diagnosis and treatment, so it is crucial to strengthen the screening and early diagnosis of these potential patients in order to take timely measures for intervention and treatment. The U.S. CDC recommends that all adults aged 18 and older be screened for hepatitis B at least once in their lifetime. Preliminary screening should include combined testing for hepatitis B surface antigen (HBsAg), hepatitis B surface antibody (anti-HBs), and hepatitis B core antibody (anti-HBc). At the same time, it is recommended that all pregnant women undergo hepatitis B screening during pregnancy, regardless of their vaccination status or previous test history, and the screening is best completed in early pregnancy [63]. T2DM screening should also be carried out early among CHB patients to reduce the risk of comorbidities. The key to T2DM screening is to monitor whether fasting blood glucose and HbA1c levels are abnormal [68]. In addition, some studies have proposed a risk stratification standard based on the “burden” of metabolic dysfunction, which classifies CHB patients into those with comorbid diabetes, comorbid overweight/obesity, or two other comorbid types of metabolic abnormalities. Drawing on this concept of risk-based management, CHB patients with comorbid diabetes or multiple other metabolic abnormalities should be given corresponding enhanced monitoring plans [58].

### 3.3. Tertiary Prevention

The core of tertiary prevention is controlling blood sugar, treating hepatitis B with medication, regularly screening for HCC, and managing comorbidities and malignant progression.

Although there are currently no specific guidelines for the management of CHB patients with T2DM, studies have shown that intensive blood glucose control may help improve clinical outcomes in these patients [26]. Optimal glycemic control, defined as maintaining HbA1c < 7% for more than 80% of the follow-up period, was associated with a significantly reduced risk of HCC in patients with CHB and T2DM (adjusted HR = 0.671, 95% CI 0.465–0.969, *p* = 0.033) [69]. The traditional treatment plan for blood sugar control in T2DM patients starts with lifestyle interventions, gradually transitions to oral hypoglycemic drugs, and as the disease progresses, most patients eventually need to start insulin [70]. Lifestyle interventions mainly focus on reducing sugar intake. Clinical observations have confirmed that replacing high-glycemic-index carbohydrates with low-glycemic-index carbohydrates in a mixed diet can significantly improve blood glucose control in patients with T1DM and T2DM. Currently, the following hypoglycemic drugs are widely used in diabetes treatment worldwide: insulin; biguanides; sulfonylureas; meglitinide derivatives; α-glucosidase inhibitors; thiazolidinediones; glucagon-like peptide-1 agonists; glucose-dependent insulinotropic polypeptide agonists; dipeptidyl peptidase IV inhibitors; and selective sodium-glucose co-transporter-2 inhibitors [65].

For patients diagnosed with hepatitis B, current liver association guidelines mainly recommend two treatment strategies: one is pegylated interferon α-2a, administered for a fixed 48-week course, which aims to achieve clinical cure by stimulating the body’s immune system to clear the virus; the other is long-term treatment with recommended nucleotide analogs (including first-line drugs entecavir, tenofovir disoproxil fumarate, and tenofovir alafenamide), which control the disease by strongly inhibiting viral replication [62].

In addition, for HBV-infected individuals who have not started treatment, regular HCC screening should be conducted to assess and control disease progression at an early stage until spontaneous HBsAg clearance is achieved. For HBeAg-positive patients, HBeAg status should be rechecked every 6–12 months. For HBeAg-negative patients with low HBV DNA levels, HBV DNA and ALT levels should be checked every 3 months for 1 year to confirm inactive disease, after which monitoring can be done every 6–12 months. Furthermore, HBsAg clearance should be checked once a year. If conditions allow, for HBeAg-negative patients with HBV DNA < 2000 IU/mL, annual quantitative HBsAg measurement may help with HCC risk stratification and optimizing monitoring plans. In addition, consider a non-invasive assessment of liver fibrosis every 2–3 years. The following populations should undergo ultrasound ± alpha-fetoprotein (AFP) testing every 6 months: patients with cirrhosis, HBsAg-positive individuals at high risk despite a non-cirrhotic liver (including Asian or Black men over 40 years old, Asian women over 50 years old, those with a first-degree relative with a history of HCC, or patients co-infected with HDV); even after HBsAg clearance, the following patients still require regular screening: patients with cirrhosis, those with a first-degree relative with HCC, or those with long-term infection (men over 40 years old, women over 50 years old) [62]. Notably, most individuals with T2DM present with obesity, and excess abdominal fat impairs the diagnostic performance of ultrasound for HCC. Thus, CT/MRI combined with AFP, or even percutaneous needle biopsy, is warranted for HCC screening and verification [71].

It is worth noting that for diabetic patients, some antidiabetic drugs have shown potential efficacy in preventing HCC and improving liver function, but the optimal medication regimen still requires further investigation. Some studies have evaluated certain antidiabetic drugs [72], the use of metformin can reduce the risk of HCC by 20%. Based on preclinical and clinical studies, multiple pleiotropic and specific anticancer mechanisms of metformin have been discovered. Metformin’s blood sugar-lowering effect is mainly attributed to its inhibition of hepatic gluconeogenesis, which is closely related to its regulation of the redox state of hepatocytes [73]; activating AMPK is one of the core aspects of its anti-tumor effect. Metformin activates AMPK through two main pathways: Metformin accumulates in mitochondria, inhibits respiratory chain complex I, reduces ATP production, lowers the ATP/AMP and ATP/ADP ratios, and activates the energy sensor AMPK; low-dose metformin interacts with Axin and LAMTOR1, activating AMPK through the lysosomal pathway. Activated AMPK phosphorylates and activates cAMP-specific phosphodiesterase 4B, reducing cAMP levels, inhibiting fructose-1,6-bisphosphatase, and thereby suppressing gluconeogenesis. In addition, AMPK-mediated inhibition of fatty acid synthesis activates hepatic fatty acid oxidation by directly phosphorylating acetyl-CoA carboxylase (ACC1/ACC2), reducing lipid storage in hepatocytes and enhancing hepatic insulin sensitivity. AMPK activation also affects the mTOR signaling pathway, exerting antiproliferative effects and inhibiting cancer cell growth [74,75]. Metformin has also been shown to promote the activation of NRF2, which is a key regulator of the antioxidant response and has been observed in animal models of T2DM or hepatotoxicity. AMPK can activate NRF2 either by directly phosphorylating NRF2 or by inhibiting GSK3β, an inhibitory regulator of NRF2 [73,76]. Metformin may exert a preventive effect in CHB patients with T2DM through the mechanisms mentioned above, but more clinical studies are still needed to verify its exact effect and mechanism in reducing the risk of HCC.

SGLT2 inhibitors are a class of chemical drugs used to treat T2DM, with additional cardiovascular and renal protective effects. For example, dapagliflozin can lower glucose levels, as well as promote weight loss, reduce blood pressure, provide cardiovascular benefits, and protect the kidneys [77,78]. Existing evidence suggests that SGLT2 inhibitors primarily reduce the risk of HCC through the indirect contribution of ‘blood sugar lowering’ itself, but the ‘drug itself’ also has direct anticancer potential through pathways such as AMPK activation, and the evidence for the latter is still accumulating. Specifically, previous studies have shown that the glucose-lowering effect of SGLT2 inhibitors is independent of insulin, but rather works by inhibiting glucose reabsorption in the proximal renal tubules and promoting urinary glucose excretion, thereby reducing plasma glucose levels. Recent studies have shown that SGLT2 inhibitors can promote AMPK phosphorylation, and AMPK is activated through phosphorylation of Thr172 in the α subunit to maintain glucose homeostasis [79]. Animal experiments have shown that such inhibitors may have beneficial regulatory effects on NAFLD/NASH; multiple clinical trials have also confirmed that SGLT2 inhibitors can improve indicators such as liver enzymes, BMI, blood lipids, blood glucose, and IR in patients with NAFLD. Therefore, this type of drug shows promising prospects in delaying the progression of liver damage in these patients [80].

Semaglutide is a long-acting glucagon-like peptide-1 receptor agonist (GLP-1RA) that promotes endogenous insulin secretion in a glucose-dependent manner, inhibits glucagon secretion, and can also act on the hypothalamic feeding center to suppress appetite and delay gastric emptying [81]. Animal studies have shown that GLP-1RA can reduce liver lipid accumulation by activating the autophagy-lysosome pathway, lower liver lipid content in high-fat-fed mice, and also improve liver function [82]. GLP-1RAs are believed to exert hepatoprotective effects through multiple mechanisms, including reducing liver steatosis by improving insulin sensitivity and decreasing de novo lipogenesis; exerting anti-inflammatory effects by inhibiting NF-κB and reducing oxidative stress; modulating the gut-liver axis by altering gut microbiota composition and intestinal permeability; and promoting weight loss and improving glycemic control. Preclinical data suggest that the hepatoprotective effects of GLP-1RAs are most pronounced in the early stages of fibrosis [83,84].

The above-mentioned medications have also shown consistent efficacy in patients with comorbidities. A cohort study conducted an in-depth evaluation of the efficacy of SGLT, and the results indicated that in patients with comorbid T2DM and CHB, the use of SGLT2 inhibitors can reduce the risk of HCC [85]. Another study based on real-world cohorts found that for CHB-related liver cancer patients with diabetes who underwent radical resection, the use of metformin significantly improved overall survival (OS) and progression-free survival (PFS) [86]. In addition, some studies suggest lowering the threshold for starting antiviral therapy in CHB patients with comorbidities in clinical practice, and adopting a combined treatment approach with potent antivirals and management of metabolic dysfunction; however, specific implementation still requires further investigation [87].

## 4. Discussion and Perspectives

This review systematically summarizes the existing evidence from epidemiology and molecular mechanisms, to clinical management, aiming to provide a comprehensive theoretical basis and practical guidance for HCC risk prevention and control in patients with comorbidities. However, this study also has certain limitations. In core areas such as the mechanisms by which T2DM and CHB synergistically promote cancer and clinical management strategies, because there is currently a lack of direct evidence, the related comorbidity mechanisms and management strategy recommendations presented in this paper are summarized based on existing fragmented evidence. Future research, both basic and clinical, is needed to confirm these findings.

Although the aforementioned drugs (Metformin, SGLT2, GLP-1Ras) have shown potential in preclinical and clinical studies to reduce the risk of HCC in CHB patients with T2DM, the existing evidence has multiple limitations. Observational studies face issues such as indication bias, insufficient adjustment for antiviral therapy, and lack of detailed data on liver disease severity and metabolic phenotypes, which limit the reliability of causal inferences.

In terms of mechanisms and treatment, existing evidence suggests several clues worth noting. The coexistence of T2DM is an independent risk factor for impaired antiviral efficacy in CHB patients, and good glycemic control helps improve virological response, suggesting that early intervention in T2DM may enhance the effectiveness of antiviral therapy [88]. Metabolomics studies have shown that the levels of isoleucine, histamine, and tryptophan continuously decrease during the progression from CHB to HCC. Of particular note is histamine—as a key pro-inflammatory factor, it plays an important role in various inflammatory diseases and may be involved in liver damage and malignant transformation by mediating inflammatory responses [89]. SGLT2 inhibitors have shown significant protective effects against cirrhosis and HCC in patients with chronic viral hepatitis combined with diabetes. Their mechanism may be related to improving metabolism, reducing inflammation, and inhibiting the progression of fibrosis. Although the specific mechanisms and long-term effects still need further clarification, this finding suggests that SGLT2 inhibitors may become a potential therapeutic option for preventing cirrhosis and HCC in patients with chronic viral hepatitis and metabolic diseases, especially those with long-term diabetes [90]. Emerging inflammatory mediators represented by NETs, as factors promoting the liver tumor microenvironment, also provide new directions for future mechanism research and intervention targets. In summary, future research should focus on integrating the cross-regulatory mechanisms of metabolic disorders, inflammatory microenvironments, and anti-diabetic treatments, further validating the preventive potential of drugs such as SGLT2 inhibitors, and providing a theoretical basis for establishing precise mechanistic networks and effective intervention strategies.

## Figures and Tables

**Table 1:** Summary based on the source of research cohorts: cohorts of only T2DM, only CHB, and meta-analysis. aHR: adjusted Hazard Ratio; RR: Risk Ratio; HR: Hazard Ratio.

Source of Evidence	Main Research Conclusions	Risk Estimate/Effect Size	References
Cohorts with T2DM alone	T2DM are associated with an increased risk of liver cancer, and there is a correlation between HbA1c and the risk of HCC.	aHR 1.37	[15]
Cohorts with CHB alone	(1) CHB patients have a higher risk of developing diabetes compared to non-CHB patients, and different hepatitis B virus infection statuses are gradiently associated with the risk of developing T2DM;	RR 1.18	[18]
	(2) T2DM is independently associated with the risk of HCC in patients with CHB.	HR 2.08	[24]
CHB-T2DM comorbidity cohort	In CHB patients with T2DM, poor blood glucose control is significantly associated with the progression of liver fibrosis, and optimizing blood glucose management and the use of hypoglycemic drugs may slow the progression of liver fibrosis.		[25]
Meta-analysis	(1) The risk of developing HCC in T2DM patients is higher than in non-diabetic populations;	RR 1.86–2.75	[14]
	(2) Comorbidity of CHB and T2DM significantly increases the risk of HCC in patients.	RR 1.26–1.37	[20,21]

**Table 2:** Summary of the proposed mechanisms, level of evidence, and sources of evidence.

Mechanism Category	Specific Mechanistic Summary	Level of Evidence	Evidence Type (Specific to CHB-T2DM/Inferred from Related Fields)
1. T2DM aggravates CHB progression			
Metabolic disorders & lipid accumulation	IR can lead to an overload of free fatty acids (FFA) and hepatic steatosis by enhancing lipolysis and promoting de novo lipogenesis in the liver; this process may synergistically promote HBV replication and exacerbate liver fibrosis.	Moderate	Inferred (T2DM/MASLD + CHB studies) The specific causal chain still requires more direct evidence for verification
MASLD/MASH-driven hepatocarcinogenesis	Chronic hyperinsulinemia can activate the PI3K/Akt/mTOR pathway, promoting abnormal hepatocyte proliferation; lipotoxic intermediates can induce endoplasmic reticulum (ER) stress and oxidative stress, leading to HBV DNA damage.	Moderate	Inferred (MASLD/HCC + diabetic metabolism) Direct verification required for comorbidity of CHB and T2DM
Oxidative stress & mitochondrial dysfunction	Hyperglycemia can induce the formation of AGEs, leading to ROS accumulation and oxidative stress, which in turn causes DNA damage.	Moderate-High	Inferred (T2DM oxidative stress + CHB hepatocarcinogenesis)
Chronic inflammation & pathway activation	Oxidative stress can activate the NF-κB pathway, inducing the release of inflammatory factors such as TNF-α and IL-6, forming a persistent inflammatory microenvironment; this process synergizes and amplifies bidirectionally with HBV infection and T2DM metabolic disorders, potentially promoting persistent HBV infection, exacerbating liver fibrosis, and increasing the risk of hepatocellular carcinoma.	Moderate-High	Inferred (general inflammation-oncology + CHB) This mechanism is mostly inferred from single-disease studies and still requires further validation.
Immune dysfunction	Hyperglycemia and oxidative stress may impair innate immunity and T cell immune function, potentially reducing the HBV clearance ability in CHB patients, thereby promoting persistent viral infection; this process is synergistic with the immunosuppression induced by HBV itself.	Moderate	Inferred (diabetic immunometabolism + CHB immunity) More direct clinical studies on comorbidity of CHB-T2DM are needed.
2. CHB disrupts glucose homeostasis & promotes T2DM			
Hepatic metabolic dysfunction	HBV infection and related liver damage can potentially induce insulin resistance and hyperglycemia by reducing glycogen synthesis and decreasing insulin clearance ability, thereby increasing the risk of developing T2DM.	Moderate	Inferred (CHB liver metabolism + T2DM pathogenesis) More direct evidence is needed for verification
HBx-mediated gluconeogenesis	The HBx protein may promote hepatic glucose production by regulating gluconeogenesis-related pathways; meanwhile, HBV-induced liver injury can reduce glycogen synthesis and decrease insulin clearance, synergistically triggering insulin resistance and hyperglycemia.	Moderate	Specific to HBV (HBx models) + inferred to T2DM
Inhibition of insulin signaling	In vitro studies have shown that HBx can upregulate PEPCK/G6Pase through activation of the NO/JNK/FOXO1 pathway, potentially promoting hepatic gluconeogenesis.	Moderate	Inferred (viral-immune crosstalk + islet dysfunction) Further in vivo studies in CHB patients are needed for verification.
Pancreatic β-cell damage	HBV infection may induce insulin resistance by inducing SOCS expression and inhibiting IRS phosphorylation, and damage pancreatic β cells through the JAK/STAT3 pathway, reducing insulin secretion.	Moderate	Inferred (HBV extra-pancreatic tropism + diabetic β-cell failure) More clinical evidence is needed
3. Synergistic carcinogenesis of T2DM + CHB			
Metabolic-lipid vicious cycle	A few studies suggest that HBV can colonize pancreatic tissue, inducing local inflammation and damaging β cells, potentially leading to impaired insulin secretion; this process forms a bidirectional vicious cycle with the metabolic disorders of T2DM, synergistically worsening liver damage and metabolic abnormalities.	Moderate	Directly proposed for comorbidity
Oncogenic signaling activation	Hyperinsulinemia/IGF-1 may synergize with HBx protein to activate the PI3K/Akt/mTOR pathway, promoting abnormal hepatocyte proliferation and anti-apoptosis, thereby increasing the risk of HCC.	Moderate	Inferred (metabolic oncogenic signaling + HBV) Direct validation of comorbidity cohorts is required.
Synergistic oxidative stress	The accumulation of AGEs/ROS related to T2DM, together with mitochondrial damage and endoplasmic reticulum stress induced by HBV/HBx, leads to oxidative stress overload and genomic instability, potentially promoting malignant transformation of hepatocytes.	Moderate-High	Inferred (dual oxidative stress sources) The clinical comorbidity-specific evidence for this mechanism still needs to be supplemented.
Inflammatory cascade amplification	Chronic inflammation related to T2DM interacts with and mutually reinforces the immune dysregulation induced by CHB, forming a persistent inflammatory microenvironment that may exacerbate progressive liver damage and promote the occurrence of liver fibrosis and HCC.	Moderate-High	Inferred (inflammatory networks in comorbidity) More comorbidity-specific research validation is needed
NETs-mediated pro-tumor microenvironment	Metabolic stress and inflammation may induce NETosis, thereby promoting Treg/Th17 polarization, potentially leading to immunosuppression, liver fibrosis, and angiogenesis.	Moderate	Inferred (MASH-HCC + neutrophil biology) Direct evidence of CHB-T2DM comorbidity still needs to be supplemented.
4. Epigenetic mechanisms			
HBV cccDNA epigenetic regulation	HBx protein can regulate the chromatin state of HBV cccDNA by recruiting epigenetic enzymes such as HATs and DNMTs, promoting persistent viral transcription.	High	Specific to HBV Evidence is sufficient in in vitro models, but the synergistic effect of T2DM in clinical comorbidity needs further study.
T2DM-induced epigenetic remodeling	Hyperglycemia and lipotoxicity may lead to DNMT/TET/HDAC/EZH2 dysregulation, which in turn causes abnormal expression of IR-related genes.	Moderate	Inferred (diabetic epigenetics + liver metabolism) The synergistic regulation of CHB-T2DM comorbidity needs to be validated.
5. Non-coding RNA/imprinted loci			
ncRNA-mediated metabolic reprogramming	ncRNAs such as miRNAs, lncRNAs, and circRNAs can participate in hepatocyte metabolic reprogramming by regulating glycolysis, lipid metabolism, mitochondrial function, and related pathways.	Moderate	Inferred (cancer metabolism ncRNA literature) The specific effects of comorbidity need further exploration.
Imprinting disturbance (IGF2/H19 LOI)	Loss of imprinting (LOI) of genes such as IGF2/H19 may lead to dysregulation of ncRNA expression, thereby disrupting metabolic homeostasis and potentially contributing to the transformation of metabolic diseases into cancer.	Low-Moderate	Inferred (imprinting-cancer-metabolism axis) Insufficient direct evidence

## Data Availability

No new data were created or analyzed in this study. Data sharing is not applicable to this article.
